# A new phosphorylated form of Ku70 identified in resistant leukemic cells confers fast but unfaithful dna repair in cancer cell lines

**DOI:** 10.18632/oncotarget.4735

**Published:** 2015-08-13

**Authors:** Julien Bouley, Lina Saad, Romain Grall, Amelie Schellenbauer, Denis Biard, Vincent Paget, Sandrine Morel-Altmeyer, Olivier Guipaud, Christophe Chambon, Bernard Salles, Karim Maloum, Hélène Merle-Béral, Sylvie Chevillard, Jozo Delic

**Affiliations:** ^1^ Laboratoire de Cancérologie Expérimentale, Institut de Radiobiologie Cellulaire et Moléculaire (IRCM), Commissariat à l’Energie Atomique et aux Energies Renouvelables (CEA), 92265 Fontenay aux Roses, France; ^2^ Institut de Maladies Emergentes et des Thérapies Innovantes (iMETI), Service d’Étude des Prions et des Infections Atypiques (SEPIA), CEA, 92265 Fontenay aux Roses, France; ^3^ Service de Spectrométrie de Masse, INRA Theix, 63122 St Genès Champanelle, France; ^4^ UMR 1331 TOXALIM, INRA/INP/UPS, F-31027 Toulouse, France; ^5^ Service d’Hématologie Biologique, Hôpital Pitié-Salpêtrière, 75000 Paris, France; ^6^ Université Pierre et Marie Curie, Paris VI, INSERM, UMR-S 872, Programmed Cell Death and Physiopathology of Tumor Cells, Centre de Recherche des Cordeliers 75000 Paris, France; ^7^ Laboratoire de Spectrométrie de Masse, Stallergens, 92160 Antony, France; ^8^ Laboratoire de Radiopathologie et de Thérapies Expérimentales, Institut de Radioprotection et de Sureté Nucléaire (IRSN), 92265 Fontenay aux Roses, France

**Keywords:** phospho-Ku70, c-NHEJ, DNA repair kinetic, CLL, gamma-H2AX/ATM/DNA-PKcs

## Abstract

Ku70-dependent canonical nonhomologous end-joining (c-NHEJ) DNA repair system is fundamental to the genome maintenance and B-cell lineage. c-NHEJ is upregulated and error-prone in incurable forms of chronic lymphocytic leukemia which also displays telomere dysfunction, multiple chromosomal aberrations and the resistance to DNA damage-induced apoptosis. We identify in these cells a novel DNA damage inducible form of phospho-Ku70. *In vitro* in different cancer cell lines, Ku70 phosphorylation occurs in a heterodimer Ku70/Ku80 complex within minutes of genotoxic stress, necessitating its interaction with DNA damage-induced kinase pS2056-DNA-PKcs and/or pS1981-ATM. The mutagenic effects of phospho-Ku70 are documented by a defective S/G2 checkpoint, accelerated disappearance of γ-H2AX foci and kinetics of DNA repair resulting in an increased level of genotoxic stress-induced chromosomal aberrations. Together, these data unveil an involvement of phospho-Ku70 in fast but inaccurate DNA repair; a new paradigm linked to both the deregulation of c-NHEJ and the resistance of malignant cells.

## INTRODUCTION

The widespread heterogeneous clinical course of chronic lymphocytic leukemia (CLL) relies on the variable extent of the genetic alterations that are related to chemorefractoriness and disease progression [1]. Historically, the first pathway to be identified as defective in CLL was the apoptotic death pathway, and this dysregulation still remains a paradigm of CLL maintenance [2, 3]. This defective apoptosis dogma has persisted even when the homeostatic balance or imbalance in the dynamic interplay between proliferation and cell death has been proposed as a new hallmark of stable (indolent) or progressive forms of CLL, respectively [4]. However, the mechanisms responsible for inducing a switch to the aggressive forms of this disease remain unclear. Aggressive CLL develops in one third of patients who then succumb rapidly due to the lack of effective therapies. Front-line treatments using alkylating agents or purine nucleoside analogues mediate cell death through DNA damages, including double strand breaks (DSBs), and p53-dependent apoptosis [5, 6]. The loss of the p53 protein, or of the ATM (ataxia telangiectasia mutated) gene product which acts upstream of p53, has been associated with drug resistance and shortened survival times [6, 7]. Recently, Notch signaling was proposed as a responsible p53-inducible antiapoptotic mechanism that should be considered in future apoptotic targeting [8, 9].

We have previously reported that 20% of CLL cell samples are resistant to DNA damage-induced apoptosis (R-CLL), irrespective of their p53 status, whilst the remaining cases have p53wt-expressing sensitive cells (S-CLL) [2]. Accelerated DNA repair and the accumulation of chromosomal aberrations have been observed in R-CLL [10]. Moreover, the upregulation of nonhomologous end-joining (NHEJ), in R-CLL was found to be error-prone *in vitro* and thus potentially mutagenic *in vivo* [11, 12]. These findings were concomitant with a telomeric dysfunction with increased Ku70 co-localization, increased level of DSBs and multiple chromosomal aberrations occurring in an R-CLL subset [13, 14]. Based on these results, we hypothesized that the resistance of malignant cells to genotoxic stress-induced apoptosis is specific to a new subset of DNA repair-related disease that is p53-independent and that may depend on a delay in the persistence of DNA damage signaling. The potential impact of such resistance upon the onset of malignancy is likely to be increased by the fact that on the resulting block on apoptosis induction may contribute to the emergence of additional resistant clones from a proliferative pool of mutant cells.

Ionizing irradiation- and cytotoxic drug-induced DSBs, including those caused by fludarabine, are repaired mainly by NHEJ which is the major cell cycle-independent repair pathway for this type of DNA damage in mammalian cells [15–19]. More recent discoveries have proposed the existence of two distinct NHEJ pathways acting with fast or slow kinetics, with different efficiencies and accuracy of the final repair product, and that are dependent on different factors [20–24]. The central player in classical NHEJ (c-NHEJ) is certainly the DNA-PK trimer containing the Ku70/Ku80 heterodimer that acts as a scaffold for the recruitment of core or processing factors, DNA-PKcs and Artemis, that further recruit the ligation Cernunos(XLF)/XRCC4/LigaseIV complex [25–27]. In addition, a phosphorylation cascade may facilitate the fine-tuning of the various stages of this repair process [28]. However, although DNA-PKcs may potentially phosphorylate nearly all members of the NHEJ complex, only its auto-phosphorylation regulates NHEJ activity [24, 25, 29]. As the overactivation of NHEJ activity in R-CLL is correlated with enhanced DNA end-binding of Ku70/Ku80 heterodimer without an increase in its expression [11], we next hypothesized that the post-translational modifications (PTMs) of Ku may be a critical step in the development of aggressive forms of CLL.

In this context, we investigated the presence of PTMs on the Ku heterodimer combining high-resolution 2D-gel electrophoresis (2D-PAGE) and mass spectrometry (MS) analysis of CLL proteins. These approaches allowed us to identify the phospho-ser27-Ku70 overexpressed in the resistant form of CLL. Further, from 2D-PAGE data analyses (pI displacements), phosphatase λ and/or irradiation treatments, the highly conserved proximal serine residue between species, serine-33 was deduced as a second site of phosphorylation occurring concomitantly with serine-27. Monoclonal antibodies, produced in mouse hybridoma cells, revealed that Ku70 phosphorylation occurs within minutes of genotoxic stress and involves DNA-PKcs and/or ATM kinase activities. By using specific vectors enabling the simultaneous shRNA-mediated inhibition of endogenous Ku70 and the expression of exogenous Ku70 resistant to shRNA (*i.e.* S27-S33-Ku70 and A27-A33-Ku70 expressing cells), we showed that phospho-Ku70 contributes to faster but error-prone DNA repair resulting in higher levels of chromosomal breaks. The persistence of this new form of Ku70 and the convergence of its putative functions underline a new paradigm for c-NHEJ regulation, which is involved in DNA damage repair and in observed instability in cancer cells.

## RESULTS

### Identification of a phosphorylated form of Ku70 in chemoresistant leukemia cells

**Figure 1:**
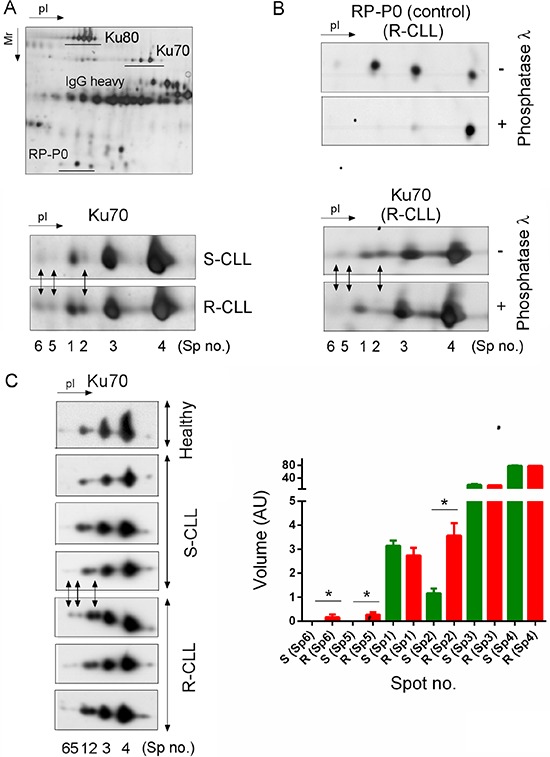
Preferential Ku70 phosphorylation in resistant CLL cells **A.** Ku heterodimer was immunopurified using Ku80 monoclonal antibodies followed by 2D-PAGE separation. The enlarged region of the 2D gel image shown reveals Ku70 protein spots that increased in abundance in R-CLL by at least two-fold in comparison with S-CLL (Spots no. 2, 5 and 6). Spot numbers refer to different isoelectric forms of Ku70. R-CLL, resistant CLL cells; S-CLL, sensitive CLL cells. **B.** Dephosphorylation experiments carried out using Ku heterodimer purified from R-CLL lymphocyte extracts. RP-P0 (belonging to heterogeneous nuclear ribonucleotide protein family, hnRNP), a phosphorylated protein co-purified with Ku heterodimer, was used as a control. **C.** Whole cell extracts of R-/S-CLL cells and healthy CD19^+^ B lymphocytes were separated by 2D-PAGE and immunoblotted against a Ku70 antibody. The images shown are representative of 6 R-CLL, 8 S-CLL and one healthy CD19^+^ B cell sample. Each bar represents the mean spot volume values (± SD) from S-CLL or R-CLL samples. AU, arbitrary unit. Mann-Whitney *U*-test, *p < 0.05.

**Figure 2:**
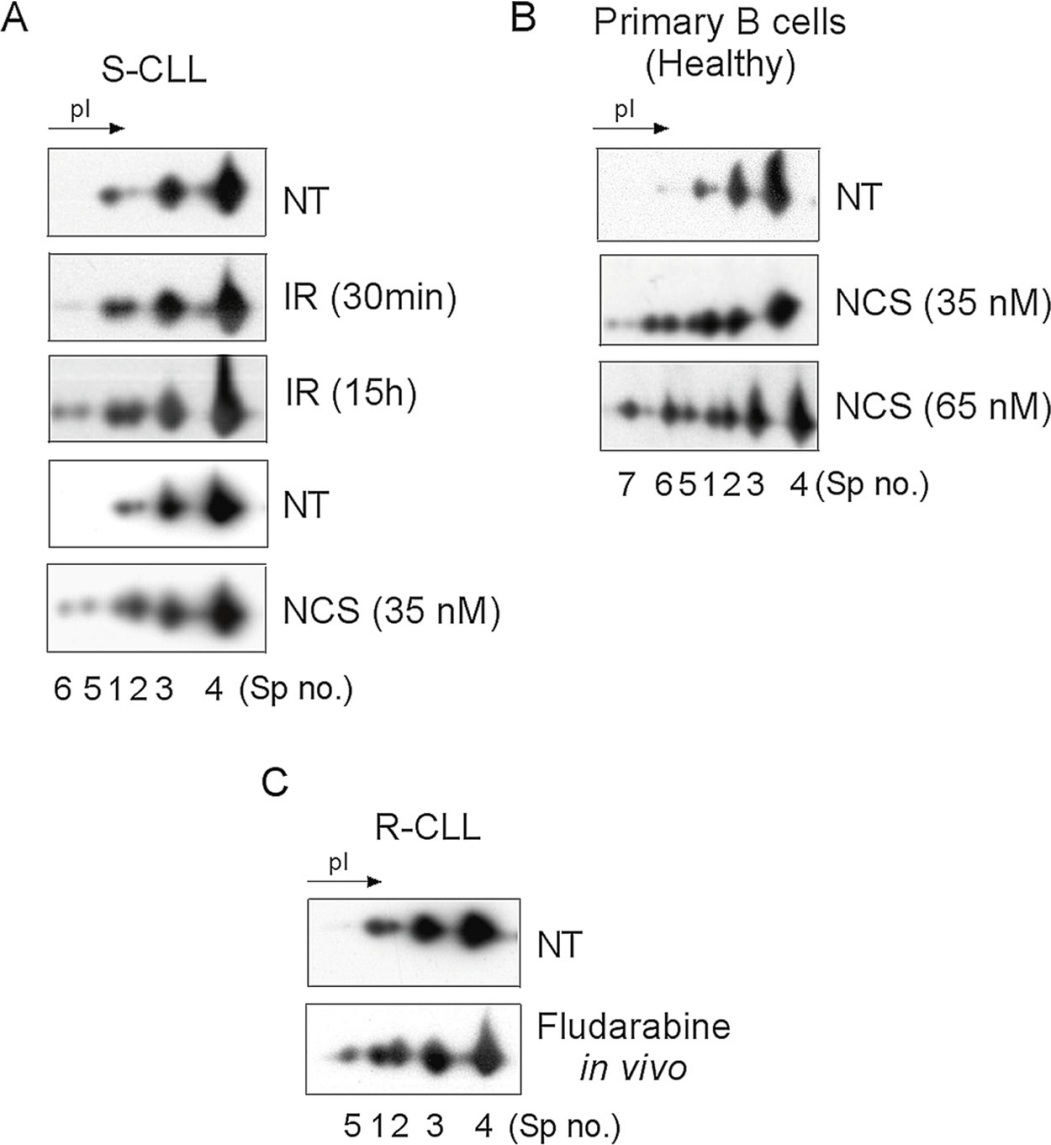
Ku70 phosphorylation after DNA DSB induction is dose- and post-treatment time-dependent in cellulo and *in vivo* following fludarabine treatment Ku70 was monitored in whole cell extracts of S-CLL cells **A.** or healthy CD19^+^ cells **B.** Cells were either untreated (NT), subjected to IR (10 Gy) or treated with NCS and harvested either 30 min or 15 h later for IR-treated cells and 30 min later only for NCS-treaterd cells. **C.** Phosphorylation of Ku70 occurs *in vivo* following exposure of CLL cells to fludarabine. Ku70 was monitored in whole cell extracts from R-CLL cells derived from a patient in the course of a fludarabine therapy.

We exploited the high-resolution potential of 2D-PAGE to compare the PTM of the Ku heterodimer between two subgroups of CLL defined by their sensitivity or resistance to DNA damage-induced apoptosis and ability to upregulate NHEJ (Supplementary Table S1). Ku heterodimer was purified by protein immunoprecipitation using Ku70 or Ku80 monoclonal antibodies followed by 2D-PAGE (Figure 1A). The different forms of Ku70 and Ku80 present in S-CLL cells were resolved, respectively, as four spots (spots N° 1, 2, 3 and 4) and at least six spots with similar molecular weights but different isoelectric points (pI). In representative R-CLL cells, Ku70 isoforms were resolved as six spots, three of which were more abundant (N° 2, 5 and 6) and had a lower pI. The intensity of spot N°2 was found to be markedly increased in R-CLL cells (2- to 2.5-fold) compared with S-CLL. Phosphorylation was the principal PTM since λ-phosphatase treatment reduced the number of Ku70 spots (Figure 1B). These results were confirmed in B cells from one healthy, six R-CLL and eight S-CLL donors (Figure 1C). We further analyzed Ku70 phosphorylation *in cellulo* by inducing DSBs by ionizing irradiation (IR) or neocarzinostatin (NCS) (Figure 2A, 2B and Supplementary Figure S1). Ku70 phosphorylation was detected at higher levels 30 min after exposure to NCS in a dose-dependent manner (Figure 2B and Supplementary Figure S1). The number of S-CLL cells with characteristic apoptotic morphology reached 85% at 15 hours after IR whilst the phosphorylation of Ku70 remained still strongly induced. This suggests that apoptotic DNA fragmentation also induced Ku70 phosphorylation (Figure 2A). Similar results were observed with immunopurified Ku heterodimer (i.e., using an anti-Ku80 antibody) indicating that the IR-induced phosphorylation of Ku70 occurs in heterodimeric complexes. Notably, Ku70 phosphorylation occurred *in vivo* in patients treated with fludarabine (Figure 2C), demonstrating that Ku70 is subjected to multiple phosphorylation events following genotoxic stress both *in cellulo* and *in vivo*.

Tandem MS/MS and MS^n^ experiments identified a major site of Ku70 phosphorylation at position serine-27 (Supplementary Figure S2A). We compared the 2D gel patterns of exogenous His-Ku70 obtained from HeLa cells co-transfected with His-wtKu70 or His-Ku70-S27A in which serine-27 was replaced by a neutral alanine (Supplementary Figure S2C). As expected, the S27A substitution altered the number of spots in the gel, confirming that Ku70-spot N°2 was indeed the S27-phosphorylated form of Ku70 (pS27-K70). Of note, the alignment of sequences from diverse organisms revealed a second highly conserved serine residue at position 33, additionally highlighting that even more acidic Ku70 spots should correspond to a double phosphorylation of Ku70 (spots N°5 and 6, Figure 1 and 2).

### pS27-Ku70 is phosphorylated by PI3-K kinases, interacts with pS2056-DNA-PKcs and pS1981-ATM

**Figure 3:**
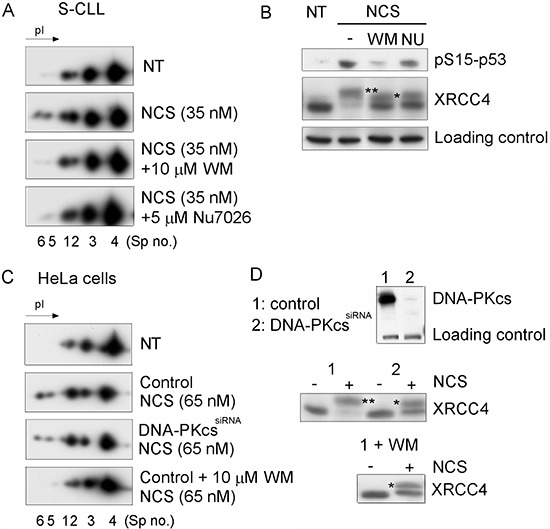
DNA DSB-induced phosphorylation of Ku70 requires DNA-PKcs activity in CLL cells **A.** S-CLL cells were either untreated (NT), treated with NCS (35 nM), pretreated for 1 h with 10 μM WM and treated with NCS, or pretreated for 1 h with 5 μM NU7026 (NU) and treated with NCS. Spot numbers refer to different isoelectric forms of Ku70. **B.** Western blot analysis showing the specificities of WM and NU treatments for the phosphorylation of p53 (pS15-p53) and XRCC4 (phosphorylated forms are labeled by asterisks). **C.** Control HeLa cells were either untreated (NT), treated with NCS alone or simultaneously treated with NCS and WM. In parallel, DNA-PKcs^Kd^ HeLa cells expressing siRNAs specific to DNA-PKcs were treated with NCS alone. Spot numbers refer to different isoelectric forms of Ku70. **D.** Western blot analysis indicating the efficiency of DNA-PKcs silencing by siRNA. The endogenous levels of DNA-PKcs and the effects of DNA-PKcs depletion, NCS and WM treatments on phosphorylation of XRCC4 were monitored.

**Figure 4:**
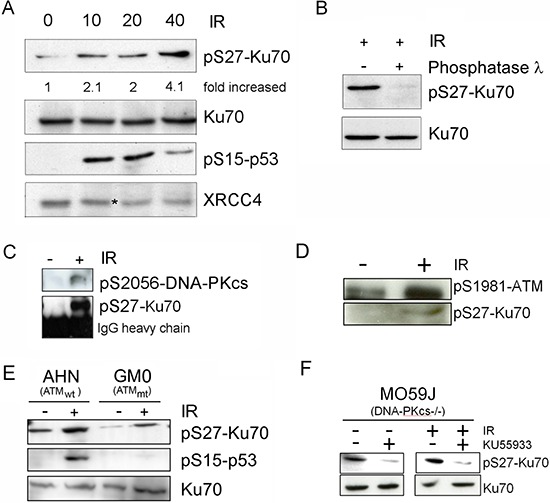
pS27-Ku70 upregulation occurs in CLL cells after IR and involves interaction with pS2056-DNA-PKcs and/or ATM **A.** HeLa cells were either left untreated (NT) or treated with 10, 20 or 40 Gy of IR and harvested 30 min later. Levels of pS27-Ku70, endogenous Ku70, phospho-XRCC4 (labeled by an asterisk) and pS15-p53 are shown. **B.** Dephosphorylation experiments were carried out using irradiated CLL proteins. **C.** pS2056-DNA-PKcs or pS1981-ATM **D.** were immunoprecipitated by anti-pS276Ku70 from CLL proteins using cells left untreated or treated with 10Gy of IR. Western blot analysis was performed with anti-pS2056-DNA-PKcs or anti-pS1981-ATM (upper panels) or anti-pS27-Ku70 (lower panels) antibodies. **E.** MO59J cell line (deficient in DNA-PKcs) were treated or not with KU55933 (10 μM) 18 h prior to 10Gy irradiation. Cells were harvested 2 h post-irradiation and Western blots probed for total endogeneous Ku70 and p-S27-Ku70. **F.** AHN (wild-type) and GM0 (GM03189 cell line expressing mutated ATM) cells were either left untreated (NT) or γ-irradiated at 10Gy and harvested 30 min later. Levels of pS27-Ku70 and endogenous total Ku70 (as control) are shown. pS15-p53 level is shown as control of a kinase deficiency in GM01389 cell line.

**Figure 5:**
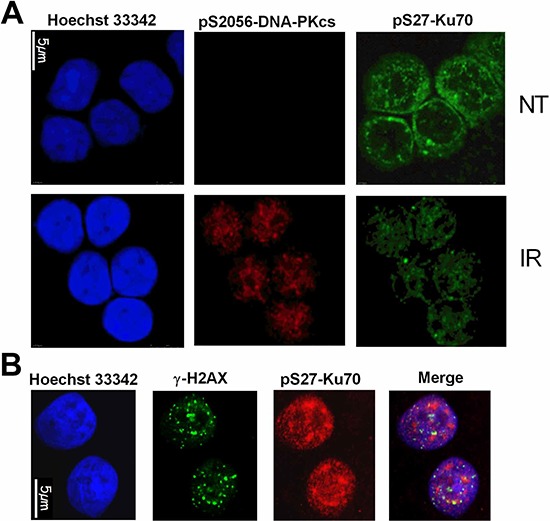
Nuclear delocalization of pS27-Ku70 following irradiation stress **A.** *In situ* immunofluorescence monitoring of pS27-Ku70 and pS2056-DNA-PKcs in resistant CLL cells left untreated (NT) or at 30min after a 10 Gy dose of IR. Nuclei were counterstained with Hoechst 33342. **B.** Simultaneous immunofluorescence labeling was proceeded following prextraction/RNase treatment procedure described in Method section. ZR75.1 cell line was irradiated at 4 Gy and following 30min of post-irradiation culture, simulataneusly labelled with anti-pS27-Ku70 (red) and anti-γ-H2AX (green). Hoechst H33342 was used for chromosomal DNA staining.

To identify the protein kinase involved in the DSB-induced phosphorylation of Ku70, we treated CLL cells (Figure 3A, 3B) and the HeLa DNA-PKcs^kd^ cell line [30] (Figure 3C, 3D) with NCS in the presence of wortmannin (WM), a PI3 kinase and PI3-like kinase inhibitor, or with NU7026, which at low concentrations inhibits DNA-PKcs but not ATM or ATR [31]. Both p53 and XRCC4 phosphorylation were used as controls [32, 33]. Our experimental conditions indicated that the phosphorylation of Ku70 is dependent on DNA-PKcs but also showed that other DNA DSB-sensing PI3-kinases, such as ATM, could also be involved in the regulation of Ku70 phosphorylation. To complement these analyses, a monoclonal antibody directed against pS27-Ku70 documented an increase of IR-induced Ku70 phosphorylation in both S-CLL (Figure 4A) and HeLa cells (data not shown). As shown in Figure 4B, pS27-Ku70 was found to be absent in irradiated protein extracts treated by λ-phosphatase, as well as in modified cell lines expressing an unphosphorylable form of Ku70 (see below). Co-immunoprecipitations using anti-pS2056-DNA-PKcs and anti-p1981-ATM antibodies demonstrated that pS27-Ku70 interacts with pS2056-DNA-PKcs following IR (Figure 4C and 4D). The experiment with PI-3K kinase deficient cell line GM03189 expressing mutated ATM, demonstrated a very weak increase in S27-Ku70 phosphorylation (Figure 4E). DNA-PKcs deficient cell line MO59J treated with ATM inhibitor KU55933 which did not exhibited an increase in S27-Ku70 phosphorilation following irradiation treatment (Figure 4F), further argues a redundant involvement of both DNA-PKcs and ATM in S27-Ku70 phosphorylation. Staining of untreated R-CLL cells showed both a cytoplasmic and nuclear distribution of pS27-Ku70 (Figure 5A), as well as a marked increase in the pS27-Ku70 level in R-CLL cells compared with S-CLL cells (immunofluorescent staining not shown). Interestingly, early after irradiation pS27-Ku70 localized only in the nuclei of irradiated R-CLL cells and appeared to colocalize with pS2056-DNA-PKcs at the sites of the DNA DSBs. In contrast, a strict colocalisation of pS27-Ku70 was not evidenced in large γ-H2AX foci covering several megabases of chromatin DNA in irradiated ZR 75.1 cells (Figure 5B). Indeed, pS27-Ku70 labelling covers nearly all chromatin area (excepting nucleoli due to the extraction RNase treatment by cytoskeleton buffer).

### pS27-S33-Ku70 contributes to faster DNA repair, a shorter S phase and greater chromosomal damage

**Figure 6:**
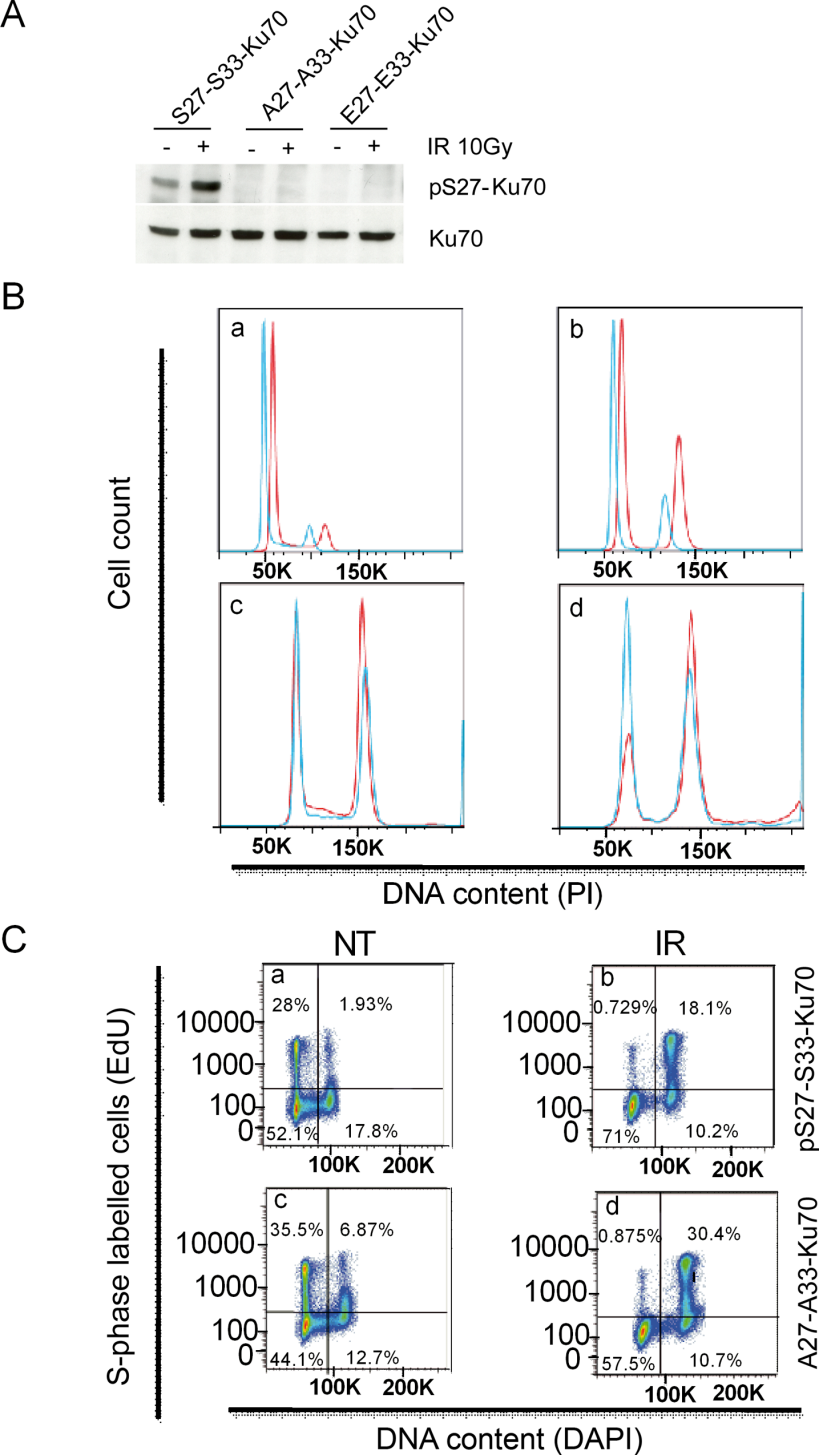

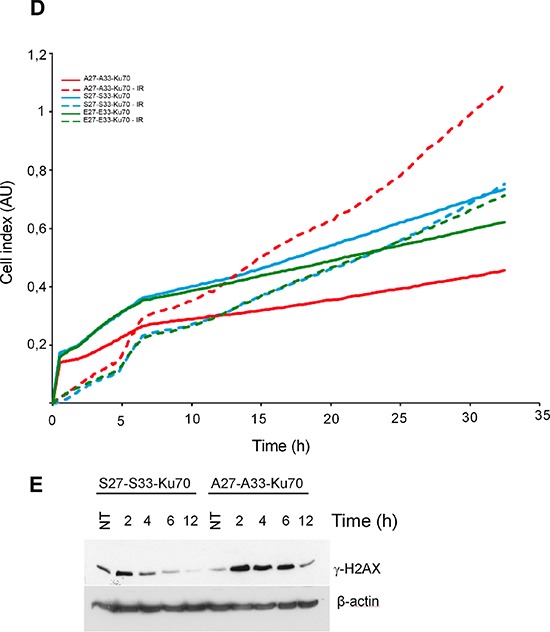

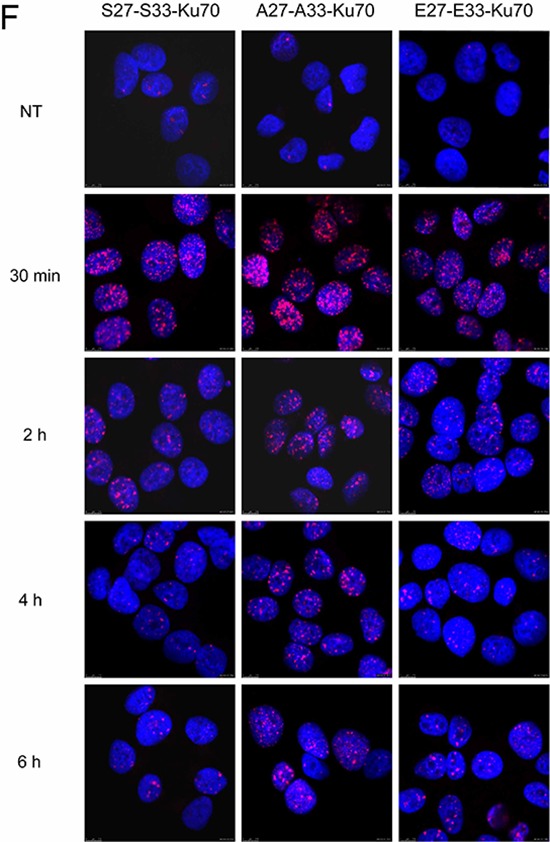

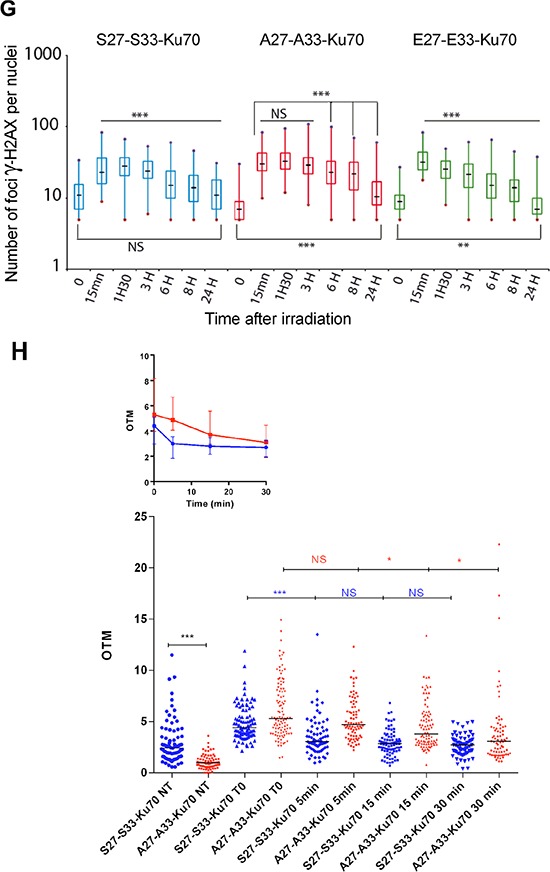

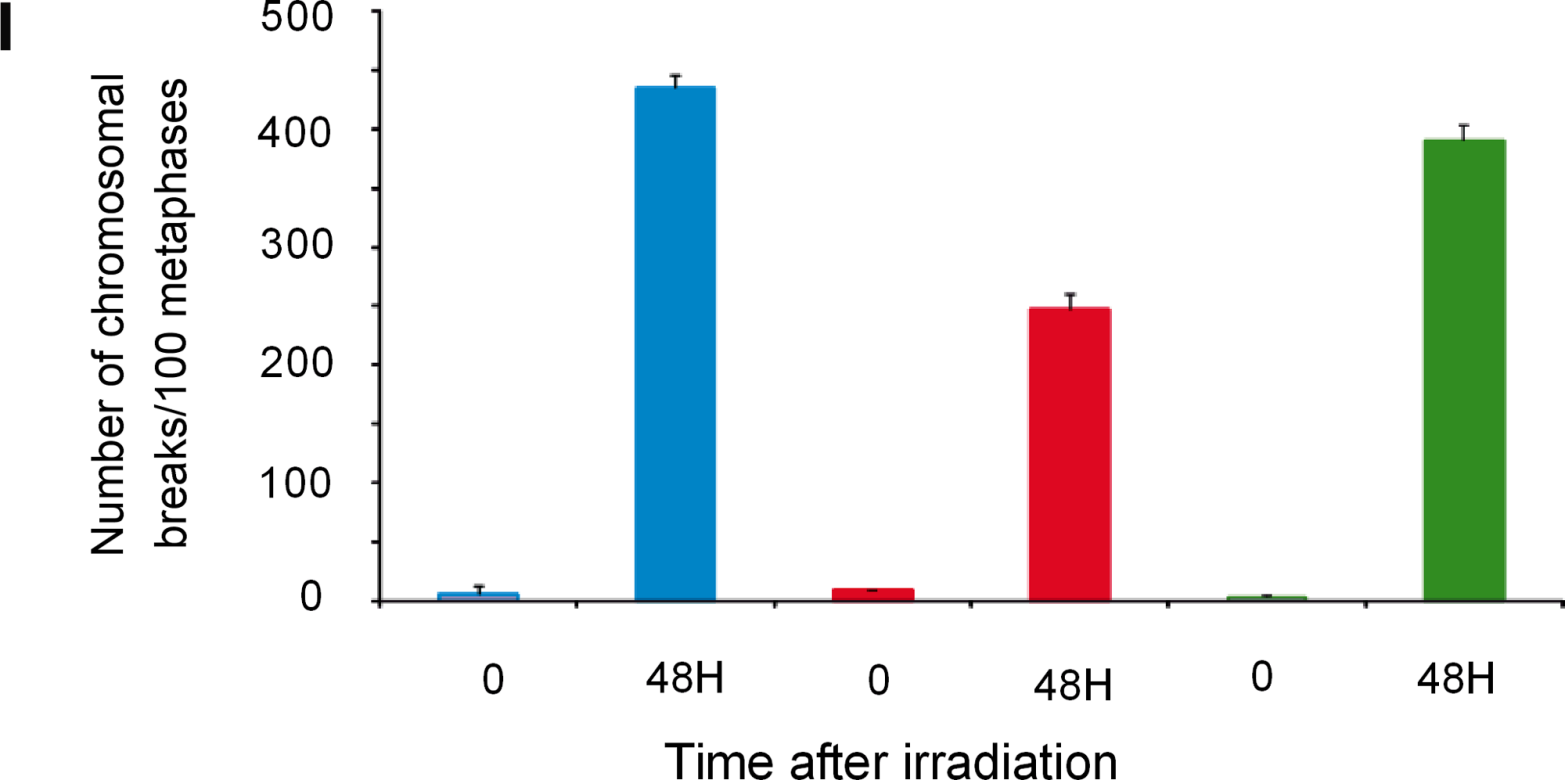
Function of the phosphorylated form of Ku70 in cell cycle checkpoint control, DNA repair and genomic stability ZR75.1 breast cancer cells express either wt-Ku70 (S27-S33-Ku70) or mutated Ku70 (A27-A33-K70 or E27-E33-Ku70). **A.** pS27-Ku70 expression was monitored using a pS27-Ku70 antibody. **B.** The cell cycle was assayed at different timepoints after IR (4Gy) in S27-S33-Ku70- (blue) and A27-A33-Ku70- (red) expressing cells E27-E33-Ku70 expressing cells shown cell cycle profile similar to S27-S33-Ku70 (not shown). The cell cycle profiles shown are of untreated cells (a), and of post-irradiated cells at 12 h (b), 24 h (c) and 48 h (d). **C.** Cell incorporation of ethynyl deoxyuridine (EdU) in S phase. Untreated (NT) or 12 h post-irradiated (4Gy) cells are shown. Cells expressed S27-S33-Ku70 (a, b) or A27-A33-Ku70 (c, d). **D.** Role of phospho-Ku70 in cell growth and proliferation following irradiation stress. The cell index (i.e. cell growth, proliferation and membrane potential) following IR (4 Gy) was defined using an xCELLigence® living cells’ real-time follow-up system (see Methods section). Full lines represent untreated cells and dashed lines indicate irradiated cells. A27-A33-Ku70- (red), S27-S33-Ku70- (blue) and E27-E33-Ku70- (green) expressing cells were evaluated. The cell index is given in arbitrary units (AU). **E.** Western blot analysis of γ-H2AX protein level following 2Gy irradiation. Cells expressing S27-S33-Ku70, A27-A33-Ku70 were untreated (NT) or harvested after indicated time post-irradiation. After PAGE of total protein extracts and transfer, the membranes were probed with anti-phospho-S139-H2AX and anti-β-actin antibodies. **F.** Kinetic of DNA damage-induced γ-H2AX, foci formation in untreated (NT) or irradiated (2Gy) cells expressing wild-type S27-S33-Ku70, mutated A27-A33-Ku70 or E27-E33-Ku70 at indicated time post-irradiation. **G.** γ-H2AX immunostaining results were analyzed in untreated and post-irradiated cells at the indicated timepoints in A27-A33-Ku70- (red), S27-S33-Ku70- (blue) and E27-E33-Ku70- (green) expressing cells. Cells exhibiting at least 5 foci per nuclei were included in this analysis. Counts were performed on at least 200 cells per condition and results are depicted as box plot distribution values [minimum (min), maximum (max), median, 25th and 75th percentiles (25th and 75th perc.)] of the number of foci obtained for each tested condition. Statistical analysis: a Wilcoxon rank test was performed. ***p < 0.0001; **p < 0.01; NS, non significant. **H.** Comet assay performed at the indicated times in S27-S33-Ku70- (blue) and A27-A33-Ku70- (red) expressing cells. An ANOVA one-way non-parametric unpaired (Kruskal-Wallis) test was applied as the statistical method for global analysis of olive tail moments (OTM); median values were calculated and presented with matched Wilcoxon values. ***p < 0.0001; **p < 0.01. Upper graph shows the median values for two cell lines with the interquantile range (Δ 75^th^ and 25^th^ percentiles). **I.** Genotoxic stress-induced chromosomal aberrations (karyotype) in S27-S33-Ku70- (blue), A27-A33-Ku70- (red), or E27-E33-Ku70- (green) expressing cells. Dicentric chromosomes due to misrepaired or unrepaired DNA DSBs were counted in at least 50 metaphases of untreated (0) or 48 h post IR (4 Gy) cells in three independent assays. Results were recalculated on 100 metaphases wirh median values and standard deviation.

We generated stable sublines from the breast cancer cell line ZR75.1, which overexpressed either the wild-type S27-S33-Ku70 protein (wt-Ku70), its mutated A27-A33-Ku70 form or the phosphomimetic E27-E33-Ku70 form, using the replicative pEBVsiKu70-CAGKu70_(R/S27-S33)_, pEBVsiKu70-CAGKu70_(R/A27-A33)_, or pEBVsiKu70-CAGKu70_(R/E27-E33)_ plasmids, respectively. We chose to study the function of Ku70 phosphorylation using breast cancer p53wt-expressing cell lines because of the absence of an established CLL-derived cell line. A particularly notable aspect of the vectors used in our experiments was their coding siRNA sequence enabling an inhibition of endogenous Ku70 (eKu70) and the expression of modified cDNA sequences of Ku70 resistant to siRNA. Efficient phosphorylation of Ku70 at serine-27 was thus only observed after IR in S27-S33-Ku70-expressing cells, demonstrating the effective inhibition of eKu70 expression, as well as the expression of mutated forms (Figure 6A).

We further investigated both cell cycle checkpoint control and S phase progression following genotoxic stress. The fraction of cells arrested in G2 phase was higher for A27-A33-Ku70- than for wt-Ku70-expressing cells (Figure 6B; E27-E33-Ku70-expressing cell data not shown), with a marked difference at 12, 24 and 48 h post-irradiation. In contrast, a faster S-phase progression was documented in wt-Ku70-expressing cells following irradiation (Figure 6C). Intrigued by the fact that A27-A33-Ku70 cells proliferate more slowly than S27-S33-Ku70 cells, we performed a real-time integrative assessment of cell proliferation, growth and membrane potential, commonly designated as a cell index or impedance [34–36]. Notably, the cell index (Figure 6D) confirmed a slower proliferation of A27-A33-Ku70-expressing cells prior to IR. Conversely, irradiated A27-A33-Ku70 cells showed higher proliferation rate than the two other cell lines tested, suggesting that the phosphorylation of Ku70 may control other cellular functions independently of DNA repair.

When the protein level (Figure 6E) or the kinetic of disappearance of γ-H2AX foci (Figure 6F) were analyzed, then both experiments indicated the higher persistence of this marker at 2 to 6 h post-irradaition in cells expressing mutated form of Ku70 as compared to wt-type or phosphomimetic form expressing cells. This was further validated by the Supplementary quantified and statistically analyzed assays (Figure 6G). Of note, cells expressing phosphomimetic form of Ku70 dysplayed an intermediate kinetic of γ-H2AX disappearance. In parallel, we probed other DNA damage associated foci dynamics in mutated- and wt-Ku70 cells (Supplementary Figure S3A and S3B). Interestingly, in these two cell lines up to 6 hrs following irradiation at 2Gy, the kinetic of p1981S-ATM foci appeared to be concomitant to these of γ-H2AX while the kinetic of 53BP1 foci occurred independently of Ku70 mutational status (Supplementary Figure S3A and S3B). Concerning pS27-Ku70 localisation, the labelling progressively widespreaded all nuclear area similarly to the labelling of total Ku70 at 2 h postirradiation (Supplementary Figure S4 upper and lower panel, respectively). Of note, at this time-point the labelling of nuclear pS2056-DNA-PKcs disappeared suggesting a putative nuclear role for pS27-Ku70 in DNA dynamic independent of DNA damage throught NHEJ. This is further supported by the increased protein level of phospho-Ku70 lately after genotoxic stress (see Supplementary Figure S4A and Figure S4B). Indeed, both ZR75.1 (Figure 4A) and Ramos cells resistant to DNA damage-induced apoptsis, after repeated exposures to lower doses of irradiation at 1Gy (a unique dose which did not induced Ku phosphorylation), per week/cell platting/passaging, exhibited an accumulation of phospho-Ku70. Also, different cancer cell lines (Supplementary Figure S4C), known to exhibit high level of genomic instability, display a variable but high level of phospho-Ku70 even in absence of exogenous genotoxic stress. Of note, BL2 lymphoblasts sensitive to apoptosis initiation by DNA damage, expresses a lower level of pS27-Ku70. We next performed comet assays to assess the DNA repair kinetics in two ZR75.1 sublines. To assess both DNA single and double strand break resolution, we used the extended alkaline lysis method [37, 38]. Notably, as estimated by the comet tail lengths, comet tail moment and olive tail moment (OTM) (Figure 6H), S27-S33-Ku70-expressing cells recovered up to 40% of their DNA damage within minutes (T0 and 5min post-irradiation, *p* < 0.0001), with nearly all DNA damage found to be repaired at 15 minutes after IR. In contrast, A27-A33-Ku70-expressing cells showed significantly higher levels of residual DNA damage and still underwent DNA damage repair at 30 min after IR. A significant difference in OTM was also observed between control cell lines, underlying higher levels of endogenous stress in S27-S33-Ku70-expressing cells. To further explore whether accuracy of DNA repair was affected in our transfected cells, we examined metaphase chromosomes after the first cell division following IR. For this purpose, colcemide-blocked metaphase chromosomes were hybridized with centromeric and PNA-telomeric probes and stained with DAPI at 48 hours after DNA damage. Dicentric chromosomes reflective of misrepaired or unrepaired DNA DSBs were counted across 50 metaphase spreads. As shown in Figure 6I, significantly higher levels of dicentric chromosomes were observed in wt-Ku70-expressing cells.

**Figure 7:**
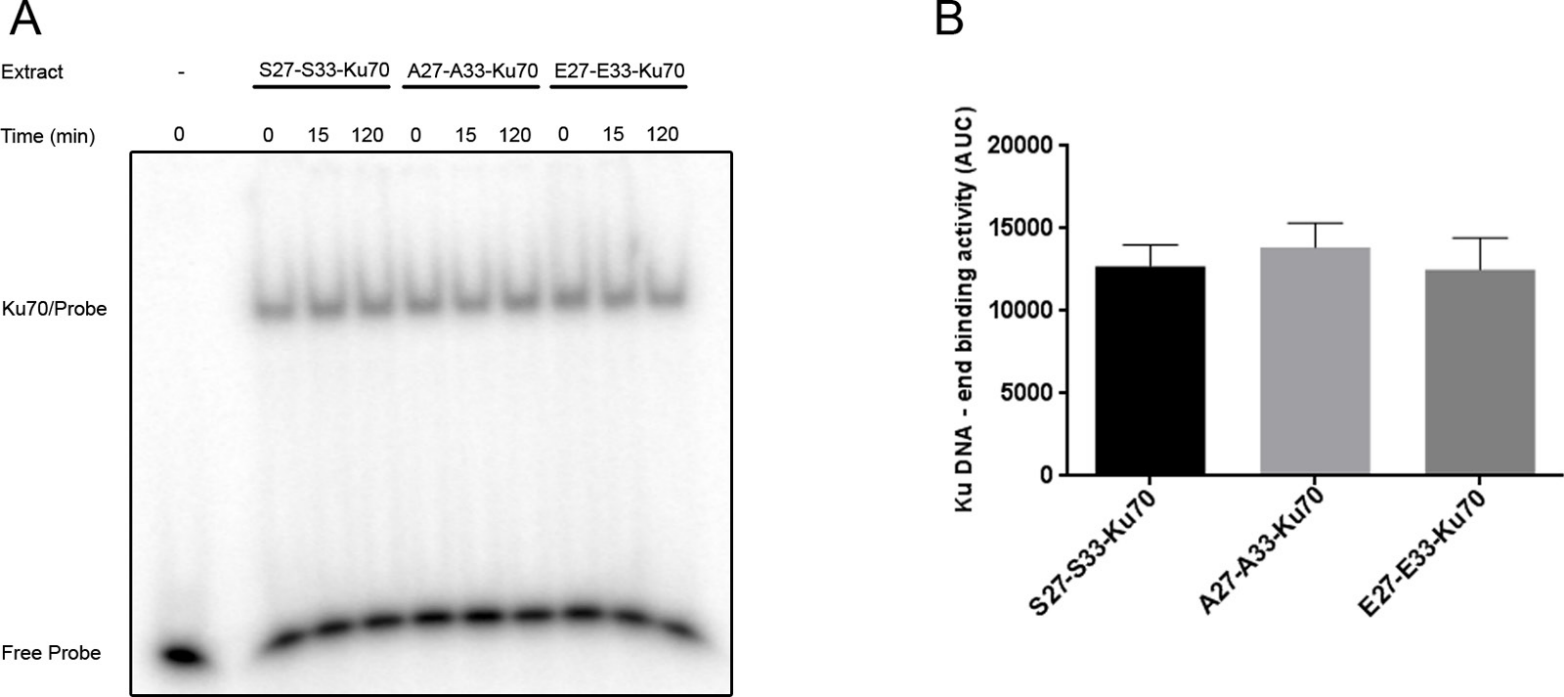
*In vitro* assay of Ku70/Ku80 heterodimer DNA-end binding activity **A.** Heterodimer Ku70/Ku80 DNA-end binding activity in protein extracts (1 μg/lane) from cells expressing S27-S33-Ku70, A27-A33-Ku70 or E27-E33-Ku70 at indicated time post-irradiation (2Gy). The results are representative of at least four independent experiments. **B.** Overall Ku70/Ku80 DNA-end binding activity in whole cell extracts of cells presented in A; AUC: air under the curve with calculated standard deviation.

To verify whether the observed acceleration of the kinetic of DNA damage repair may relay on an altered binding of Ku heterodimer to free DNA-ends, we next performed an *in vitro* assay as described elsewere [11]. Even if a weak increase in Ku DNA-end binding following irradiation was observed only in cells expressing S27-S33-Ku70 as compared to cells expressing A27-A33-Ku70 or E27-E33-Ku70 (Figure 7A), overall, there was no significant alteration in Ku DNA-end binding between proten extracts from three cell lines (Figure 7B).

## DISCUSSION

The CLL classification based on the resistance *vs.* sensitivity of the leukemic cells to DNA damage-induced apoptosis highlights a specific subgroup of this cancer which displays a DNA repair defect. This defect is strikingly correlated to the clinical features and may be further related to the appearance of new genetic and chromosomal alterations. The underlying molecular anomaly in circulating malignant cells may be the result of inefficient front-line therapy. Notably, among a large previously evaluated cohort of patients (over 300 cases [39], 15 leukemic donors treated with alkylating agents/base analogues, including fludarabine, had evolved from an indolent to more aggressive form of disease and their malignant cells had progressed from an initial apoptotic sensitivity to a tolerance of DNA damage. Here, we provide evidence that these cells overexpress a new form phospho-Ku70. Two phosphorylable serine residues on Ku70 at positions 27 and 33 were experimentally found to be important for the function of this protein in response to DNA damage. In randomly screened 14 patient CLL samples (8 S-CLL and 6 R-CLL) for which we previously established global NHEJ activity, we observed altered phospho-Ku70 expression (Figure 1C and Supplementary Table S1). Because of the current lack of CLL-derived cell lines, we mainly validated the functions of phospho-Ku70 in the breast cancer cell line ZR75.1 (expressing TP53wt) which expresses a basal level of phospho-Ku70 that is further upregulated by genotoxic stress. This further emphasizes that this new deregulation of DNA repair may be a common molecular alteration in chemoresistant cancer cells, as supported by the higher basal level of phospho-Ku70 in cancer cell lines resistant to DNA damage such as human glioma, osteosarcoma, hepatocellular carcinoma cell lines or cells derived from cancer of pancreas with a deficiency in homologous recombination repair (Supplementary Figure S4C). Although an induction of phospho-Ku70 occurs *in vitro* with high doses of photon irradiation, a single perfusion of fludarabine produced the same level of phospho-Ku70 induction *in vivo* (Figure 2C). Moreover, we repetitively irradiated ZR75.1 cells or Ramos lymphoblasts in culture at lower doses (single dose 1Gy) over 5 days or 7 weeks, respectively and observed that this fractionated exposure also induced Ku70 phosphorylation (Supplementary Figure S4A and S4B). This finding may be relevant in explaining why certain patients treated with low doses of alkylating and/or base analogues agents (*i.e.* chlorambucil, fludarabine) evolved to a more aggressive disease expressing phospho-Ku70.

We find that the higher level of pKu70 protein in malignant cells induced by genotoxic agents *in vivo* contributes to a higher rate of DNA repair, a lower susceptibility to apoptosis and is likely to be the source of resistance to therapeutic treatments based on DNA damaging drugs. In agreement with this, the inhibition of NHEJ DNA repair can activate the apoptotic death pathway and block cancer progression [40]. Thus, systematic screening of the constitutive levels of phospho-Ku70 in CLL patients who have never been treated could potentially be a very useful marker for clinicians to determine if front-line therapy should be replaced by other treatments (such as immunotherapy). Varible but high constitutive levels of pS27-Ku70 in cell lines derived from different type of clinically resistant and instable cancers, further support above hypothesis.

Resistant CLL has been shown to exhibit upregulated and potentially mutagenic NHEJ [10–12]. In our current study, we show that the phosphorylation of Ku70 on two new sites alters the kinetics and accuracy of DNA repair. Data presented here indicate that the phosphorylation of serine-27 and serine-33 residues on Ku70 depends on the PI-3K kinase family (DNA-PKcs and ATM), resulting in a faster rate of DNA repair without affecting *in vitro* DNA-end binding activity of heterodimer Ku (Figure 7). Only the sites of autophosphorylation of DNA-PKcs have been used thus far to document the altered kinetics of NHEJ [25, 29]. That factors other than kinases may regulate the efficiency of NHEJ repair is supported by a recent study which reported the involvement of phosphatase PP4 in this type of DNA repair [41]. Fractionated irradiation *in vitro* and the persistence of phospho-Ku70 levels several months after *in vivo* administration of fludarabine, suggest an implication of responsive phosphatase deficiency in resistant cells.

The mammalian NHEJ DNA repair system, active in all phases of the cell cycle, is promoted by 53BP1, a signaling factor that impairs homologous recombination [17–19, 42, 43]. In parallel, more than 85% of irradiation-induced DSBs in the G1 and G2 phases of the cell cycle are repaired rapidly by this system [44]. Effectively, the slow component of DSB repair, which represents ~15% of irradiation-induced DSBs, includes homologous recombination but also NHEJ [45]. This slow component of DSB repair has been reported to be dependent on ATM whilst fast DNA repair appears to be ATM-independent. Our current data indicating the involvement of ATM in Ku70 phosphorylation implicate ATM also in the fast component of DSB repair. Taking into account these findings and the accurate assessment of the DSB repair dynamics relevant to NHEJ activity, we have here applied three complementary methodologies (i.e. alkaline comet assay, measurement of γ-H2AX foci and assessment of chromosomal structural aberrations after the first cell division), in unsynchronized cells in culture irradiated at physiological doses. Phospho-S27-Ku70 may matche with phospho-S2056-DNA-PKcs localization at sites of DNA damage only transitory and early after genotoxic stress in primary R-CLL cells (Figure 5A) while the colocalisation with γ-H2AX was not evident in wholly irradiated cells since pS27-Ku70 progressively increased in nuclear area without clear large foci formation (Figure 5B and Supplementary Figure S3, upper panel). Even if each γ-H2AX focus may theoretically correspond to one DSBs [46], it covers the megabases of chromatin domain. Only the high-resolution microscopy coupled with laser microirradiation and specific cell treatments may allow an evidencing of Ku80 smaler foci colocalisation with some but not with all of γ-H2AX nuclear foci [47]. Experimental procedure according to this last study (i.e. the prextraction cell treatment with cytoskeleton buffer and RNase), allowed an evidencing of some chromatin regions with enriched phospho-Ku70 that may overlap with large γ-H2AX foci 30min post-irradiation (Figure 5B), that may suggest the existence of chromatine (active?) domains with “preferential” repair that may deserve further characterizations. Of note, phospho-Ku70 partner phospho-S2056-DNA-PKcs did not localized into γ-H2AX or ATM foci at 2 h postirradiation (Supplementary Figure S3A and S3B), further suggesting an involvement of phospho-Ku70 and pS2056-DNA-PKcs in fast c-NHEJ further documented here by commet assay (Figure 6H). In parallel, both γ-H2AX (Figure 6F) and pS1981-ATM (Supplementary Figure S3A and S3B) foci disappeared more rapidly in pS27-S33-Ku70 than in A27-A33-Ku70 expressing cells while the 53BP1 nuclear labelling still persisted; data which are consistent with the NHEJ promotion and the HR inhibition [43]. The data we obtained clearly converge towards an upregulation of DSB repair in cells expressing phospho-Ku70 resulting in a higher level of chromosomal breaks. This upregulation is further supported by the cell cycle checkpoint activation and the S phase progression observed in these cells. Concerning phosphomimetic form of Ku70, inermediate cell responses were observed in both kinetic of γ-H2AX foci and the number of dicentric chromosomes. For this reason we focused other analyses on alanine- and serine-form of Ku70 only. Current data indicate effectively that phosphomimetic form of Ku70 may alter interaction of Ku70 with specific nuclear partners (data not shown).

In light of the threshold of DSBs necessary to trigger G2/M arrest in mammalian cells [48], our current data indicate that mutant A27-A33-Ku70 protein may brake DNA repair resulting in a persistence of DSBs when compared to cells expressing phospho-Ku70. Increased levels of dicentric chromosomes that are the result of chromosome breaks due to misrepaired DSBs [49], indicate that cells expressing phospho-Ku70 commit more final errors in DNA repair than cells expressing the unphosphorylatable form of Ku70. This cytogenetic approach appears to be the most appropriate methodology to assess the accuracy of DSB repair in living cells compared to other techniques such as pulse-field gel electrophoresis which have limited sensitivity at physiological doses of irradiation [50]. Cytogenetic analysis enables the effective detection of one dicentric chromosome that has resulted from a chromosome break due to two DSBs after G2 release [49]. This further emphasizes a putative role for wild type S27-S33-Ku70 in the temporal control of DNA repair following genotoxic stress. Accordingly, a constitutively higher level of phospho-Ku70 protein induced by genotoxic agents *in vivo* would contribute to a higher rate of DNA repair, lower susceptibility to apoptosis and may be the source of the resistance of aggressive CLL forms to therapeutic treatments based on DNA damaging drugs.

This form of Ku70 appears causative for the inaccuracy of NHEJ-mediated DNA repair that warrants further investigations. Current collaborative study through label-free protein identification/quantification by applying the orbital based mass spectrometry indicates a significant difference between protein partners interacting with Ku70 or phospho-Ku70. These partners, involved or not (directly) in DNA repair have been immunopurified with anti-pS27-Ku70 or anti-Ku70 clone N3H10 specific for aa 506–541 (data not shown). Interestingly, among known non-significantly different partners we have identified Ku80 and Ku70 it self. This may be one of explanations why *in vitro* Ku heterodimer DNA-end binding does not differ between cell sublines expressing or not phospho-Ku70 (Figure 7). Thus, further identification of factors that interact with phospho-Ku70 (and/or DNA-PK complex), should help us to better understand the fine balance that exists between fast and slow DNA repair that is critical to cancer cell fates. The existence of the phospho-Ku70 specific factors is supported by the fact that 5 hours after irradiation, A27-A33-Ku70-expressing cells exhibited a higher proliferation rate than S27-S33-Ku70-expressing cells. A cytoplasmic membrane association of Ku70 has been reported for TCF [51] and for the detoxification enzyme metalloproteinase MMP9 [52] whose expression is under β-catenin control. Ku70 may also directly control cell death inhibition by blocking Bax [53, 54] or Hdm2 [55]. In addition, several studies have implicated Ku70 in DNA structural dynamics and repair through interactions with the mismatch-repair proteins MSH6 [56], c-Myc [57], CAF-1 [58, 59], Recq1 [60] and Werner syndrome protein helicases [61] or heterochromatin protein HP1γ [62]. Our identification of new site of phosphorylation of Ku70 involving PI-3K kinase ATM opens a possible link between the fast and slow components of DSB DNA repair. In agreement with this, NBS1, which forms the MRN complex necessary for ATM activation, has already been reported as a protein interacting with Ku70 [63]. This interaction is dependent on Ku70 acetylation and subsequent release of proapoptotic Bax from Ku70-Bax complex. Thus, besides the ubiquitination, targeting Ku70 for proteasomal degradation [55], the acetylation was the only known post-transcriptional modification of Ku70 affecting its biological functions. Determining whether phospho-Ku70 is involved in these interactions would provide new insights into the link between DNA repair (both, HR and c-NHEJ) and apoptosis and their pathological deregulation.

Overall presented data suggest that pKu70 could be a critical biomarker for cancer cell sensitivity to DNA damage response whilst it obviously may convey genomic instability as a hallmark of cancer cells. In agreement with this, the involvement of c-NHEJ in chromosomal translocations associated with tumorigenesis has been shown to be strikingly restricted to human cells [64]. Depicting the molecular partners involved in fine tuning of phospho-Ku70 dynamic, would help a further understanding of the dysregulation of c-NHEJ pathway in cancer cells.

## MATERIALS AND METHODS

### Malignant B cell isolation and the assessment of cell sensitivity to DNA damage-induced apoptosis

The patients enrolled in the study were diagnosed with CLL following cytological and immunological analyses. These patients had a Matutes score of between 4 and 5, corresponding to the immunophenotyping of B cells with a low or absent expression of FMC7, CD79b, monotypic kappa or lambda light chain and expression of CD5 and CD23. CLL cell sampling was performed at least three months after chemotherapy (if any), excepting for one patient’s sample obtained before and 2 h after fludarabine treatment. Patient follow-ups were performed at the Pitié-Salpêtrière Hospital (Paris, France) between 1998 and 2012. The clinical data are presented in Supplementary Table S1. In accordance with the direction of the Pitié-Salpêtrière Hospital Ethics Committee (CCPPRB), all patients participating in this study gave written informed consent. B cells were negatively selected from fresh blood samples using the Rosettesep B-cell enrichment cocktail (Stemcell Biotechnologies, France) and isolated using a ficoll density gradient. B-cells were grown at 37°C/5% CO_2_ in RPMI 1640 medium supplemented with hepes, glutamine, and non-essential amino acids (Invitrogen, France). Apoptosis resulting from DNA damage induction and the according classification of CLL cells were assessed as previously described [11].

### Chemicals and DNA damage induction

Neocarzinostatin (NCS was a generous gift of V. Favaudon (Institut Curie, Orsay, France). NU7026 and KU55933 (Calbiochem, France) and wortmannin (WM, Sigma, France) were prepared in dry dimethyl sulfoxide and stored at −20°C. Cells were pre-treated with kinase inhibitors for 18 hours and either γ-irradiated at 10 Gy or treated with NCS.

### Cell lines, pEBV-CMVKu70 vector cloning and transfection

BL2 and Ramos (Burkitt lymphoma) EBV^−^ lymphoblasts were provided by J. Wiels (Instutute Gystave Roussy, Villejuif, France); GM03189 (Ataxia Telangiectasia, (EBV^+^) lymphoblasts were from Coriel Institute for Medical Research (NJ, USA); AHH-1 fibroblasts from healthy individual derive from W. G. Thilly lab (MIT, Cambridge, MA, USA); all other cell lines used in this study were from American Type Culture Collection (ATCC, VA, USA). BL2 and Ramos cells were grown in RPMI Glutamax medium (Life Technology, France) supplemented with 10% (vol/vol) fetal calf serum (Life Technology); Capan1 cells were grown in same medium supplemented with 20% fetal calf serum. ZR75.1 cell lines, U20S, HT29, A549, HepG2, MO59J and U373 cells were grown in DMEM Glutamax (Life Technology) medium supplemented with 10% fetal calf serum. Control and DNA-PKcs^kd^ HeLa cell lines [30] were grown at 37°C/5% CO_2_ in Dulbecco’s modified Eagle’s medium F12 (DMEM F12; Life Technologies, France) supplemented with 10% fetal calf serum (Sigma). To introduce exogenously expressed Ku70 cDNA into cells, the coding region of the human *KU70* gene (pBS-6His-Ku70) was cloned into the pcDNA 3.1 vector (pcDNA-HisKu70-S27A-S33A; S27E-S33E) (Life Technologies) and point mutations at the S27 and S33 residues were introduced using the QuickChange^TM^ site-directed mutagenesis kit (Agilent Technologies, France) to create the A27-A33 and E27-E33 mutants. These mutants were further used to construct the replicative pEBVsiKu70-CAGKu70_(R/S27-S33)_, pEBVsiKu70-CAGKu70_(R/A27-A33)_, or pEBVsiKu70-CAGKu70_(R/E27-E33)_, plasmids, respectively. pEBV-CAGKu70 constructs (wild-type or mutant forms) were transfected into ZR75.1 cells using Amaxa Nucleofector according to the manufacturer’s instructions (Lonza, France). Stable populations of cells expressing either an exogenous wild type or mutant Ku70 were established under hygromycin selection (250 μg/mL, PAA Laboratories GmbH, Austria). ZR75.1 transfected cells were then maintained in culture in DMEM GlutaMAX medium (Life Technologies) supplemented with 10% of heat-inactivated fetal calf serum and non-essential amino acids in the presence of 125 μg/mL hygromycin B.

### Analysis of the cell cycle by flow cytometry

Cells were resuspended at a density of 10^6^/ml in PBS with 5% FCS, fixed in 3 volumes of 70% ethanol (50% final concentration) and then incubated for 30 min at 4°C. The cells were subsequently centrifuged at 1200 rpm for 5 min at 4°C and resuspended in buffer containing 0.294 g citric acid, 0.716 g disodium sulfate, 10 ml distilled water and 30 ml HBSS ph 7.8 for 15 min at room temperature. After the addition of 1ml PBS/5% FCS, the cells were centrifuged at 1200 rpm for 5 min and washed 3 times in HBSS. The cells were treated with RNase A (DNase free) at a final concentration of 0.3 mg/ml in HBSS and PI was added to a final concentration of 20 μg/ml. PI fluorescence was acquired on a SORP LSR-II analyzer (BD Biosciences, San Jose, CA, ) with log acquisition (filter B685_35-A). Data were analyzed using FlowJo software (Treestar, Ashland, OR). S phase progression was assessed using “Click-it” assay (Invitrogen) according to the supplier’s instructions. DNA-intercalating analog ethynyl deoxyuridine (EdU) was detected in untreated or irradiated cells at different timepoints post-irradiation (10Gy) using the SORP LSR-II analyzer (BD Biosciences) with filters B530-30A for Alexafluor 488 or U450-50A for DAPI acquisition and subsequent analysis by FlowJo software.

### Cytogenetic analysis

Metaphase chromosomes were prepared as described previously [65]. Briefly, unexposed or 48 h postirradiated (4Gy) cells were treated with colcemide (60ng/ml, 90min) followed by 25 min of hypotonic treatment (75mM KCl). The cells were gradually fixed in methanol/acetic acid (3/1, vol/vol). Metaphase chromosome spreads were hybridized with a cyanine3-telomeric PNA probe (DAKO, France) followed by hybridization with FITC-Pan centromeric DNA probe (Cambio, Cambridge, UK). After chromosomal DNA staining with DAPI, fluorescence microscope (Nikon FXA, Kingston, UK) image acquisition and analysis was achieved using QUIPS CGH Software (Vysis, France). To asses chromosomal break counts, the following chromosomal structural aberrations were scored: dicentrics (dic), centric and acentric rings (r), and chromosome breaks (csb). Dic and r were assumed to be the result of two breaks and csb of one break.

### Comet assay

To assess the kinetics of global chromosomal (single- and double-strand) DNA repair in living cells, comet assays were performed under alkaline conditions as previously described [66]. Briefly, 1–2 × 10^4^ untreated or irradiated (4Gy) cells, after an indicated period postirradiation, were embedded in low melting point agarose (0.7% w/vol) and deposited on prepared slides precoated with two layers of agarose (1%, w/vol), and coverslipped. After 10 min incubation on ice, the coverslips were removed and the slides were placed in lysis solution (NaCl 2.5M, sodium lauryl sarcosine 0.1% (w/vol), EDTA 0.1M ph 8.0, Tris 10 mM; pH 10), overnight at 4°C. After lysis, the slides were neutralized in Tris-HCl buffer (0.4M, ph7.4) over three 10 min incubations. Prior to electrophoresis and to achieve chromosomal DNA unwinding, slides were placed in alkaline electrophoresis buffer (EDTA 1mM, NaOH 300 mM, pH13.0) for 30 min. Electrophoresis was performed at a constant 300 mA for 30 min. After neutralization (Tris 0.4 M, pH 7.4) and ethidium bromide staining, comets were assessed using an automatized Zeiss Axio (Jena, Germany) fluorescent microscope and acquisition Metasystems following analysis by Metafer4 software (MetaSystems Gmbh, Altlussheim, Germany).

### xCELLigence^®^ living cell real-time follow-up

xCELLigence^®^ instrument Real-Time Cell Analyzer (RTCA) (ACEA Biosciences, San Diego, CA), offers a means to monitor cellular responses in real time, without exogenous labellings, through impedance-based technology. The E-plates (specific 96 well microplates covered with micro-electrodes, ACEA Biosciences), allow the measures of electrical impedance that are relevant of dynamic monitoring of cell viability and cytotoxicity as well as cell proliferation. The usefulness of this system in cell biology and compatibility with ionizing irradiation were reported elsewhere [67]. The background of the E-plates was determined in 50 μl of cell culture medium and subsequently 150 μl of each cell suspension was added (3×10^3^ cells/well). Cells were incubated for 30 min at 37°C and E-plates were placed in a RTCA instrument. Cells were grown for at least 24 h, with impedance measurements performed every 5 min for 6 h (adhesion phase), then every 15 min (proliferation phase). After at least 24 h, cells were irradiated at 4 Gy and were monitored again every 5 min for 6 h (early effects), and then every 10 min for at least 24 h (late effects). Cell index (CI) raw data values were calculated by Zi-Z0 [Ohm]/15[Ohm]; where Z0 is the background resistance and Zi is the individual time point of resistance. A normalized cell index was also calculated by the software at the selected normalization timepoint, chosen as the time just before irradiation.

### Antibodies, western blotting analysis and immunofluorescence

Western blotting and fluorescent immunostaining were performed as previously described [11, 62]. Anti-Ku70 (N3H10), anti-Ku80 (111), anti-DNA-PKcs (18–2 + 25–4 + 42-psc) antibodies were purchased from Neomarkers (Thermo Scientific, France). Antibodies against phosphorylated pS15-p53 and, pS2056-DNA-PKcs (ab18192) were obtained from Cell Signaling Technology (Ozyme, France) and Abcam (Cambridge, UK), respectively. XRCC4 antibody (ab 145) was also obtained from Abcam. The Ku7390 peptide with the sequence CENLEA-pS-GDYKY was synthesized by Biosyntan GmbH (Berlin, Germany) and used for BALB/c mouse immunization and production of monoclonal antibodies against phosphorylated pS27-Ku70. Hybridoma cells were generated by fusing spleen cells from immunized BALB/c mice with the myeloma cell line SP2/0 (BioGenes GmbH, Berlin, Germany). Anti-mouse or anti-rabbit secondary antibodies were obtained from Jackson Immunoresearch Laboratories (West Grove, PA). For γ-HA2X foci assessment, cells were plated 48 h prior to treatment on Lab-Tek slides, irradiated at 2 or 4 Gy and stained at indicated times. Monoclonal anti-S139-γ-H2AX (clone JBW103) antibody was from Upstate (Merck-Millipore, France). For γ-H2AX, pS1981-ATM, 53BP1 and pS2056-DNA-PKcs foci colocalizations, antibodies used were from Upstate (Merck-Millipore), Temecula, CA (Millipore), Cell Signaling (#4937, Ozyme, France) and Abcam, respectivelly. For p27-Ku70 and γ-H2AX colocalization monoclonal rabbit anti-γ-H2AX antibody (#9718, Cell Signaling) was used according to slightly modified experimental procedure [47]. Briefly, previously described cytoskeleton buffer (CSK) contained 0.25% Tition X-100 (instead of 0.7%) and pre-extraction time was adapted to 5 min. After cell fixation (2% paraformaldehyde in PBS), cells have been incubated for 1 h at room temperature in PBS buffer containing 0.01% Triton X-100 and 10% goat serum and rabbit anti-γ-H2AX and mouse anti-phospho-S27-Ku70 antibodies. Anti-rabbit-AlexaFluor 488 and anti-mouse-Alexafluor 594 (purchased from Life Technologies) were used as secondary antibodies.

### Immunoprecipitation

For immunoprecipitation assays, anti-Ku70, anti-Ku80, anti-pS27-Ku70, anti-S1981-ATM or anti-S2056-DNA-PKcsantibodies were coupled to magnetic anti-mouse IgG Dynabeads (Life Technologies) or Bio-Estapor microspheres anti-mouse IgG (Merck Millipore), according to the manufacturer’s protocol. Protein extracts were incubated at 4°C for 2 h under gentle agitation of these beads in 20 mM Hepes (pH 7.5), 150 mM NaCl, 5% glycerol, 2, 5 mM EDTA, 0.5 mM dithiothreitol, 0.5% nonidet P-40, protease inhibitor mixture tablets (Complete Mini, Roche Diagnostics), trypsin-like protease inhibitor (Sigma) and phosphatase inhibitor cocktails (Sigma). The supernatant was removed over a Dynal MPC magnet (Life Technologies). After three washes in the same buffer, proteins were eluted using a 2D-gel rehydration buffer (see below) and stored at −80°C until use.

### Phosphatase treatment

Phosphatase treatments were performed on immunoprecipitated proteins by using λ phosphatase (New England Biolabs, Ipswich, MA) according to the manufacturer’s instructions. The control and phosphatase-treated samples were then concentrated using Microcon YM-30 centrifugal filter units (Merck-Millipore, France), analyzed by gel electrophoresis.

### 2D-gel electrophoresis

For 2D-PAGE separations, whole-cell extracts (50–75 μg proteins) or immunoprecipitated Ku proteins were resuspended in a rehydration solution containing 8M urea, 1M thiourea, 4% CHAPS, 0.012% HED solution, 0.5% IPG buffer, non linear pH 3–10, 0.25% IPG buffer, non linear pH 3–11, and 0.0001% Coomassie brilliant blue G. This solution was applied to Immobiline non linear pH 3–10NL (18 or 24 cm) DryStrip gels and separated by isoelectric focusing using the Ettan IPGphor system (GE/Amersham Biosciences, France). Isoelectric focusing was performed for a total of 37000 V to 54000 V, starting at 200 V and gradually raising the voltage to 10000 V. The strips were equilibrated in urea-containing buffer (reduction and alkylation) before loading onto SDS-polyacrylamide gels (8% acrylamide). The immunoprecipitated proteins were stained with silver nitrate, Sypro Ruby (Bio-Rad, France) or the Ku proteins were immunodetected after transfer onto PVDF membranes (Merck-Millipore).

### Mass spectrometry

Immunoprecipitated Ku70 protein from R-CLL lymphocytes was separated by 2D-PAGE and individual Ku70 spots (n°1, 2, 3 and 4) were excised from 2D-gels stained with Sypro Ruby (Life Technology), processed by tryptic in-gel digestion and analyzed by mass spectrometry (MS). Peptide samples were spun at 18000g, desalted using a C_18_ ZipTip (Merck-Millipore), according to the supplier’s instructions and analyzed by MALDI-TOF MS in a positive polarity mode (AB Sciex, France). The presence of one phosphopeptide was clearly evidenced from spot n°2. MS/MS and MS^n^ spectra from the phosphopeptide were obtained using an LTQ linear ion trap (Thermo Scientific, France).

## SUPPLEMENTARY FIGURES AND TABLE



## Abbreviations

2D: two-dimensional
ATM: Ataxia telangiectasia mutated
CLL: chronic lymphocytic leukemia
DSBs: double-strand breaks
eKu70: endogenous Ku70
IR: Ionizing irradiation
MALDI: matrix-assisted laser desorption ionization
MS: mass spectrometry
MS/MS: tandem MS
MW: molecular weight
NCS: neocarzinostatin
NHEJ: non homologous end-joining
OTM: olive tail moment
pI: isoelectric point
pS27-Ku70: S27-phosphorylated form of Ku70
PTM: post-translational modification
S: sensitive
SDS-PAGE: sodium dodecyl sulphate-polyacrylamide gel electrophoresis
TOF: time-of-flight
R: resistant
WM: wortmannin.
