# Supplementary material for: A new phosphorylated form of Ku70 identified in resistant leukemic cells confers fast but unfaithful dna repair in cancer cell lines
Source: Oncotarget. 2015 Aug 13;6(29):27980–8000. doi: 10.18632/oncotarget.4735 (PMC4695039; doi:10.18632/oncotarget.4735)
Supplement: Supplementary file 1 [file oncotarget-06-27980-s001.pdf]

SUPPLEMENTARY FIGURES AND TABLE

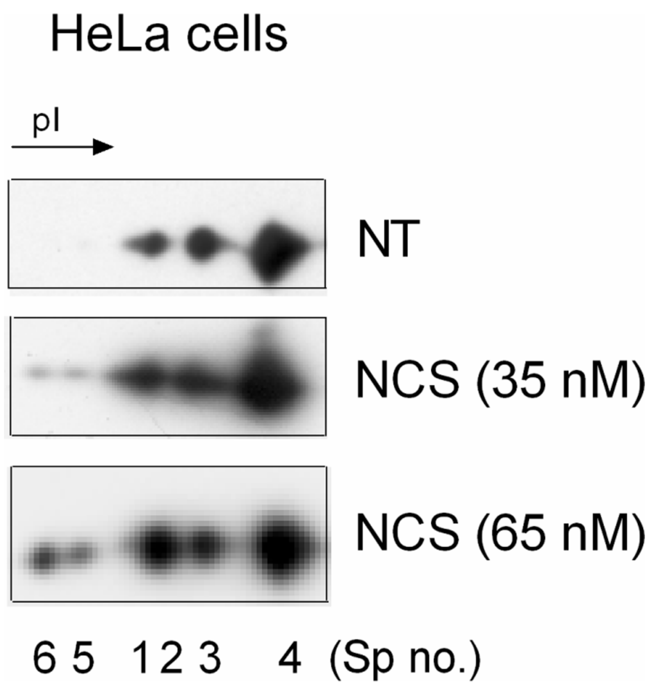

Supplementary Figure S1: Ku70 phosphorylation in HeLa cells following the induction of DNA DSBs. Ku70 was monitored by 2D-PAGE immunoblotting from whole HeLa cell extracts. Cells were either untreated (UT) or treated with NCS (35 nM or 65 nM) and harvested 30 min later. Spot numbers refer to different isoelectric forms of Ku70.

A

Ku70 phospho-site		pS27-Ku70
Tryptic peptide	K.TEGDEEAEEEQEENLEA SGDYK.Y	K.TEGDEEAEEEQEENLEAp SGDYK.Y
$M+H^+$	2500.9958 Da	2580.9621 Da
Spot no. 2	0	X
Spot no. 1, 3, 4	X	0

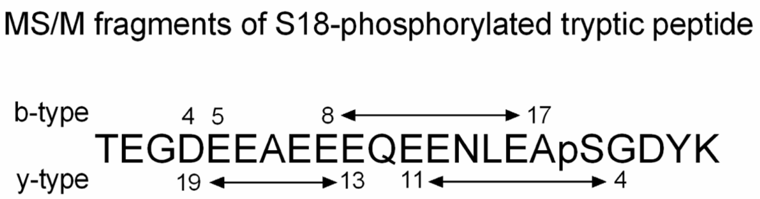

B

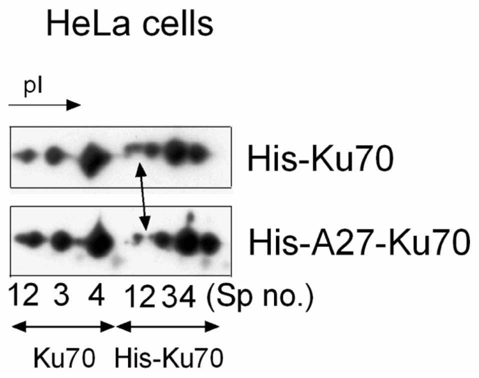

C

```
Position (Ku70_HUMAN)          S27     S33
MSGWESYYKTEGDEEAEE---EQEENLEASGDYKYSGRDSLI     P12956   Ku70_HUMAN
MSEWESYYKTEGEEEEEE---E--ESPDTGGEYKYSGRDSLI     P23475   Ku70_MOUSE
MSEWESYYKTEGEEEEEE---E--QSPDTNGEYKYSGRDSLI     E9PT85   Ku70_RAT
MANWGNYFPNEDDVDEQ-------DEEESGGDYKSSGRDSLV     A8KC26   Ku70_ZEBRAFISCH
```

Supplementary Figure S2: S27-dependent phosphorylation of Ku70 identified by 2D-PAGE and mass spectrometry. A. A monoisotopic peptide signal was detected at m/z 2501 in a tryptic peptide mixture from phosphorylated spot N°2 by MALDI-TOF-MS. The signal at m/z 2580.86 matched the theoretical m/z of phospho-Ku70$_{10-31}$ (m/z 2580.96). In tryptic peptide mixtures from nonphosphorylated spots N° 1, 3 and 4, a signal at m/z 2501 (80 U smaller) matched the theoretical m/z of Ku70$_{10-31}$ (2500.9957). **B.** A meta-stable signal, characteristic of a neutral loss of phosphoric acid (-98 U) from a phosphopeptide, confirmed the presence of phospho-Ku70$_{10-31}$ only in tryptic peptide mixture from spot N°2. This phosphopeptide was sequenced by MS-MS. A doubly charged parent ion at m/z 1291 was selected for fragmentation. The spectrum shows most of the y-type and b-type fragment ions that matched the sequence of Ku70$_{10-31}$ where S18 is phosphorylated (pS). **C.** Endogenous Ku70 and exogenous His-Ku70 were monitored by 2D-PAGE immunoblotting from whole HeLa cell extracts. Spot numbers refer to different isoelectric forms of Ku70. Note that the 3-10 pH gradient used was not linear.

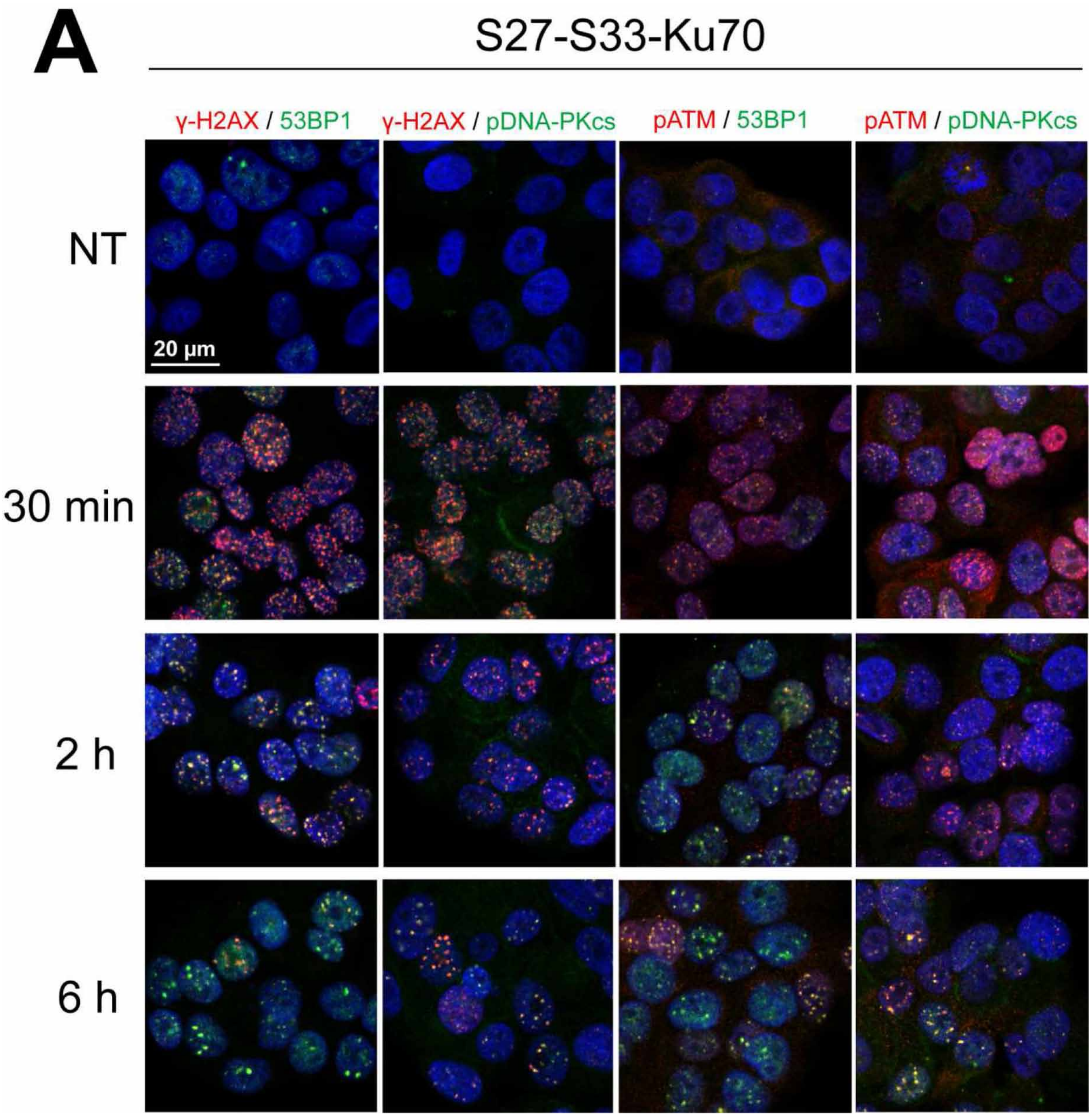

Supplementary Figure S3: Kinetic of DNA damage-associated foci in Ku70 cell sublines. Kinetic of DNA damage-induced γ-H2AX, pS1981-ATM, pS2056-DNA-PKcs and 53BP1 foci formation in cells expressing wild-type S27-S33-Ku70 A or mutated A27-A33-Ku70 B protein. (*Continued*)

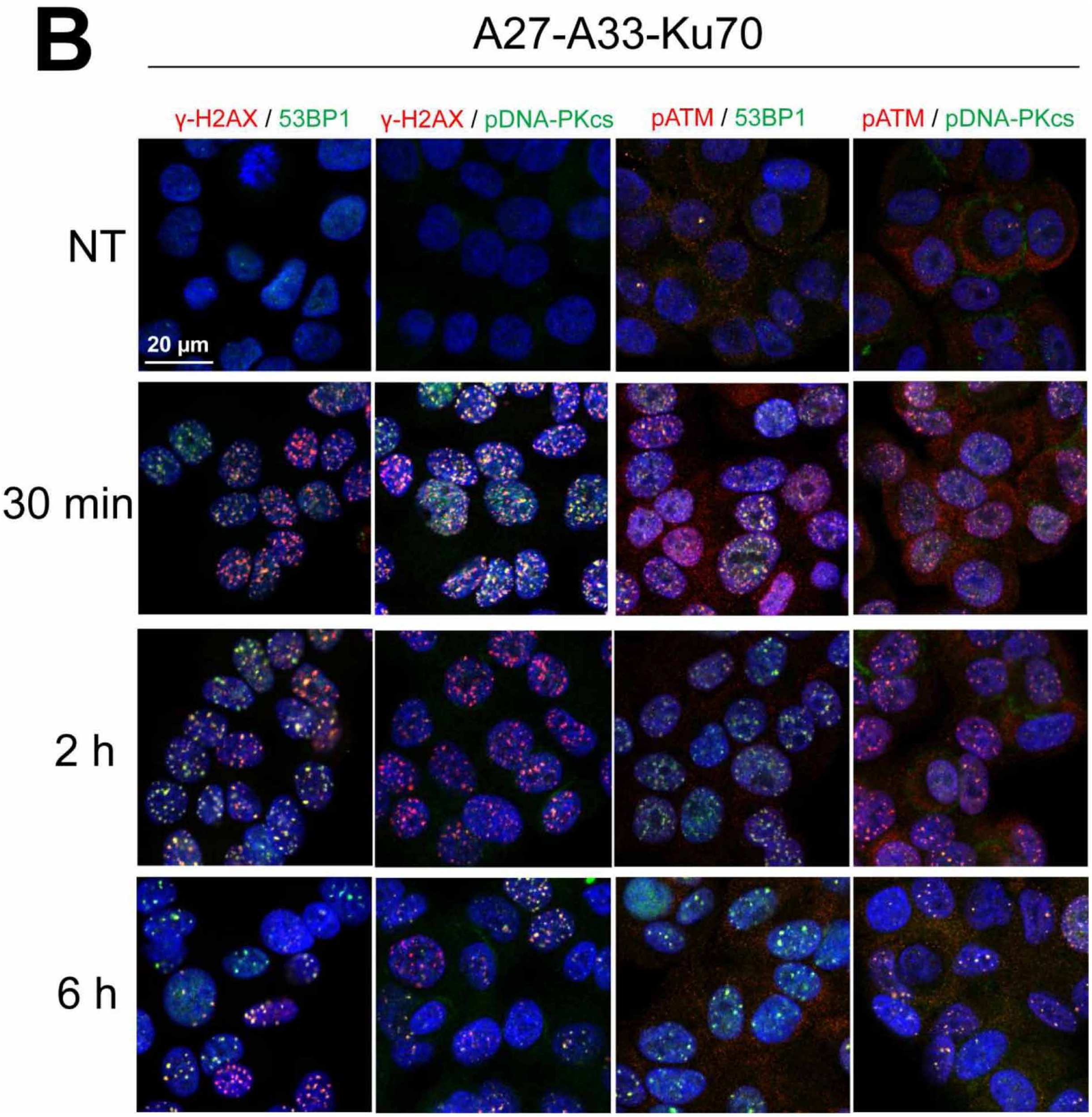

Supplementary Figure S3: (*Continued*) Kinetic of DNA damage-associated foci in Ku70 cell sublines. Double immunostainings were performed with indicated mouse monoclonal antibodies against γ-H2AX and pS1981-ATM (red), and rabbit polyclonal 53-BP1 and phospho-S2056-DNA-PKcs antibodies (green). Immunostaining results were analyzed in untreated (NT) and post-irradiated (2Gy) cells at the indicated time points. Only two antibodies' pairing merged images with nuclear counterstaining (Hoechst 33342) were shown in cells expressing S27-S33-Ku70 or A27-A33-Ku70. (*Continued*)

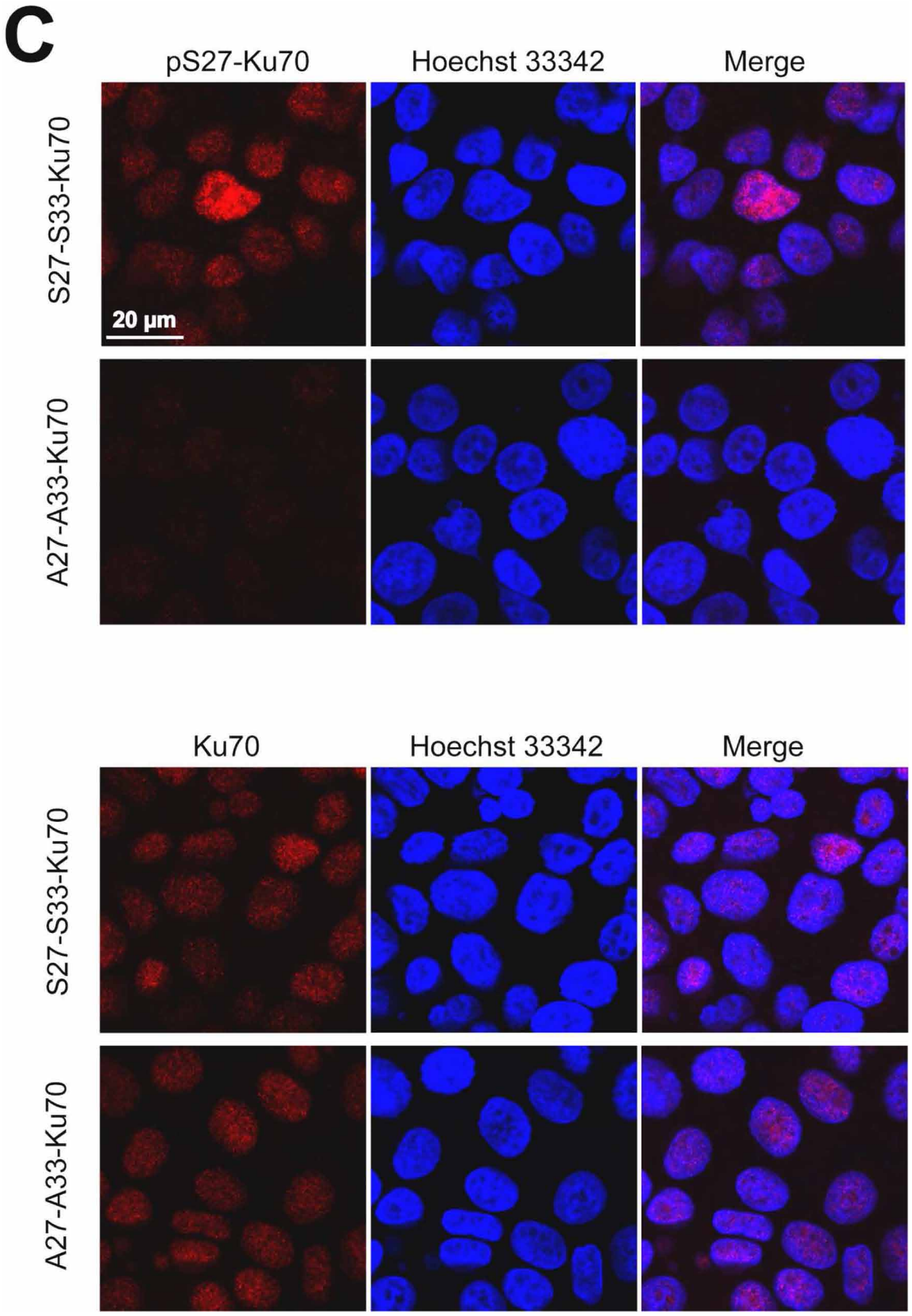

Supplementary Figure S3: (*Continued*) Kinetic of DNA damage-associated foci in Ku70 cell sublines. C. Classical immunofluorescence detection of pS27-Ku70 in cell sublines expressing wild-type or mutated Ku70.pS27-Ku70 (upper panel) or total Ku70 (lower panel) immunostainings at 2h post-irradiation (2Gy) in cells expressing wild-type (S27-S33-Ku70) or mutated Ku70 (A27-A33-Ku70). Cells were fixed for 15 min in formaldehyde solution (2% in PBS w/o Ca, Mg) and permeabilized in 0.1% TritonX-100 for 10 min. Unspecific antibody binding was prevented by an incubation for 60min at room temperature in 10% goat serum. Ku antibodies (clone N3H10 or anti-pS27-Ku70), and secondary antibody anti-mouse alexa32, were incubated in same buffer (containing 10% goat serum).

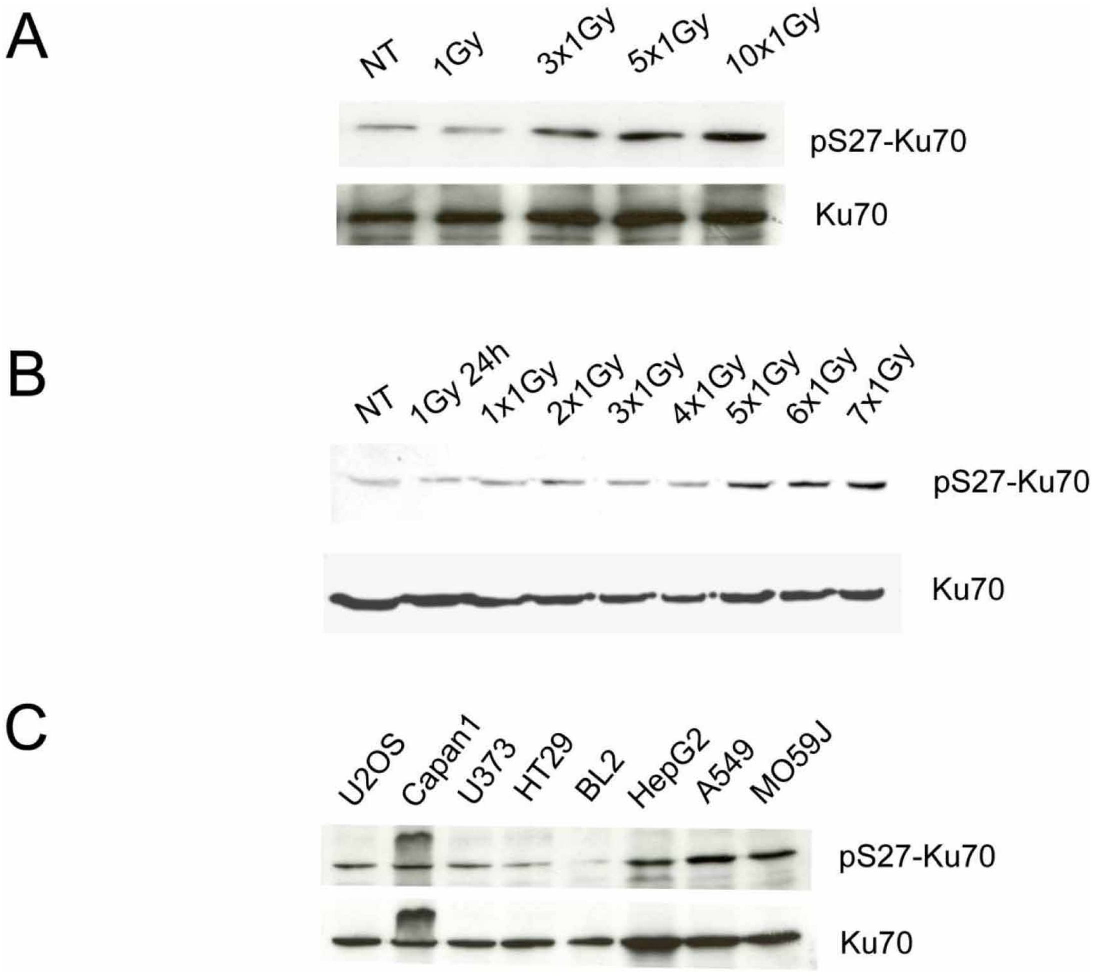

Supplementary Figure S4: pS27-Ku70 protein expression level (Western blots) in cancer cell lines exposed or not to fractionated ionizing irradiation. A. Breast cancer cell line ZR75.1 was untreated (NT) or irradiated before plating (two plating/week) once, three, five or ten times at 1Gy and lysed 24h after each irradiation and probed for pS27-Ku70 expression by Western blot. **B.** Burkitt lymphoma Ramos cell line (expressing p53mt and resisting to DNA damage-induced apoptosis), was untreated or irradiated at 1Gy on each plating once per week during 7 weeks and probed for pS27-Ku70 expression before each irradiation/plating. **C.** Constitutive phospho-Ku70 expression level in human osteosarcoma (U2OS), pancreas adenocarcinoma (Capan1), glioma (U373, MO59J), colorectal carcinoma (HT29), Burkitt lymphoma (BL2), hepatocarcinoma (HepG2) and lung adenocarcinoma (A549) cell lines.

Supplementary Table S1: Clinical characteristics and outcomes of CLL patients analyzed in this study.

Patient N°	Sex	Age	Matutes score	Treatement received	Apoptosis *in vitro* after γ-IR (10 Gy)	TP53	IgVH muta-tional status	NHEJ overactivity after γ-IR	Ku70 phos-phor-ylation	Apoptosis (%) UT	Apoptosis (%) IR
**S1**	M	58	4	-	Sensitive	wt	mut	-	-	27	99
**S2**	M	85	5	-	Sensitive	wt	mut	-	-	5	90
**S3**	M	81	4	-	Sensitive	wt	mut	-	-	4	69
**S4**	F	76	5	Fludarabine*	Sensitive	wt	mut	-	-	3	91
**S5**	M	75	5	-	Sensitive	ND	mut	-	-	10	90
**S6**	M	82	5	-	Sensitive	ND	mut	-	-	4	85
**S7**	F	61	5	-	Sensitive	wt	mut	-	-	13	77
**S8**	F	85	5	-	Sensitive	ND	ND	-	-	9	70
**R1**	F	90	5	Alkylants	Resistant	wt	ND	+	+	2	7
**R2**	M	57	4	Alkylants/ Campath	Resistant	wt	unmut	+	+	10	10
**R3**	F	93	5	Alkylants	Resistant	wt	mut	+	+	7	9
**R4**	M	81	5	Alkylants/ Fludarabine	Resistant	mut 220	mut	+	+	7	8
**R5**	F	75	4	Fludarabine*	Resistant	wt	unmut	+	+	5	9
**R6**	F	74	5	Fludarabine	Resistant	del/ mono	mut	+	+	3	3

The Matutes score is based on the immunophenotypic analysis of 5 markers, giving a value of 1 for each marker according to whether it is typical of CLL i.e. $CD5^+$, $CD23^+$, $FMC7^-$, and $CD79b^-$, shows weak expression of monotypic kappa or lambda light chain. M, male; F, female and age is expressed in years. Treatments (if any) were received at least 3 months before cell sampling for DNA extraction with the exception of one patient who received fludarabine during course of this study (Figure 2c). In this patient, cell sampling occurred before and immediately after fludarabine treatment. An apoptosis score was established 24 h after cell exposure to 10 Gy of ionizing radiation. The somatic mutational status of immunoglobulin heavy-chain variable genes (IgVH) has been established according to the 98% homology cut-off value to the closest germ-line gene; i.e. less than 98% homology was considered to be mutated (M), whereas a 98% or greater homology was considered to be unmutated (Praz et al., 2008). TP53 status was examined by FISH using LSI ATM/LSI p53 probe sets (Abbott, France) which are complementary to the 11q22.3 and 17p13.1 genomic regions and by a search for mutations as described previously (Masdehors et al. 2000). ND, not determined; del, deletion. NHEJ activity was established as previously described (Deriano et al, 2005, 2006). The Ku70 phosphorylation status was defined in this study.
